# Estimating healthcare resource needs for COVID-19 patients in Nigeria

**DOI:** 10.11604/pamj.2020.37.293.26017

**Published:** 2020-12-02

**Authors:** Adeteju Ogunbameru, Kali Barrett, Arinola Joda, Yasin Azim Khan, Petros Pechlivanoglou, Stephen Mac, David Naimark, Raphael Ximenes, Beate Sander

**Affiliations:** 1 Institute of Health Policy, Management and Evaluation, University of Toronto, Toronto, Ontario, Canada,; 2 Toronto Health Economics and Technology Assessment (THETA) Collaborative, University Health Network, Toronto, Ontario, Canada,; 3 University Health Network, Toronto, Ontario, Canada,; 4 Department of Clinical Pharmacy and Biopharmacy, Faculty of Pharmacy, University of Lagos, Lagos, Nigeria,; 5 Child Health Evaluative Sciences, The Hospital for Sick Children (SickKids) Research Institute, Toronto, Ontario, Canada,; 6 ICES, Toronto, Ontario, Canada,; 7 Sunnybrook Health Sciences Centre, Toronto, Ontario, Canada,; 8 Escola de Matemática Aplicada, Fundação Getúlio Vargas, Rio de Janeiro, Brazil,; 9 Public Health Ontario, Toronto, Ontario, Canada

**Keywords:** Coronavirus, infectious diseases, simulation model, public health, health care, Nigeria

## Abstract

**Introduction:**

continuous assessment of healthcare resources during the COVID-19 pandemic will help in proper planning and to prevent an overwhelming of the Nigerian healthcare system. In this study, we aim to predict the effect of COVID-19 on hospital resources in Nigeria.

**Methods:**

we adopted a previously published discrete-time, individual-level, health-state transition model of symptomatic COVID-19 patients to the Nigerian healthcare system and COVID-19 epidemiology in Nigeria by September 2020. We simulated different combined scenarios of epidemic trajectories and acute care capacity. Primary outcomes included the expected cumulative number of cases, days until depletion resources and the number of deaths associated with resource constraints. Outcomes were predicted over a 60-day time horizon.

**Results:**

in our best-case epidemic trajectory, which implies successful implementation of public health measures to control COVID-19 spread, assuming all three resource scenarios, hospital resources would not be expended within the 60-days time horizon. In our worst-case epidemic trajectory, assuming conservative resource scenario, only ventilated ICU beds would be depleted after 39 days and 16 patients were projected to die while waiting for ventilated ICU bed. Acute care resources were only sufficient in the three epidemic trajectory scenarios when combined with a substantial increase in healthcare resources.

**Conclusion:**

substantial increase in hospital resources is required to manage the COVID-19 pandemic in Nigeria, even as the infection growth rate declines. Given Nigeria's limited health resources, it is imperative to focus on maintaining aggressive public health measures as well as increasing hospital resources to reduce COVID-19 transmission further.

## Introduction

The World Health Organization declared the spread of severe acute respiratory syndrome coronavirus 2 (SARS-CoV-2) a Public Health Emergency of International concern on January 30^th^, 2020 and later a pandemic on March 11^th^, 2020 [1]. Africa recorded the first case of coronavirus disease 2019 (COVID-19) on February 14^th^, 2020, in Egypt [2] and since then has reported over 1,072,858 cases and 22,440 deaths as of September 3^rd^, 2020 [3]. Nigeria reported the first case of COVID-19 on February 29^th^, 2020 [4]. By September 4^th^, 54,588 confirmed cumulative cases had been reported [4]. The average daily growth rate of cumulative cases in Nigeria from when 100 cases were reported on March 29^th^, 2020 (which correspond to the date lockdown was initiated in the most affected states) to September 3^rd^, 2020 is 4.1% [4]. While the lockdown of the three most affected states in the country was lifted on May 4^th^, 2020, the mean growth rate from May 5^th^, 2020 to September 3^rd^, 2020 declined to 2.5% [4]. From June 1^st^, 2020, to September 3^rd^, 2020, the mean growth rate furthered decreased to 1.8% [4].

The daily incidence and case-fatality rates of COVID-19 in Nigeria and other African countries have been low since the start of the pandemic compared to what was observed between March and May in Europe, China and North America [3,5]. However, the spread of COVID-19 among African countries occurred rapidly in the early days of COVID-19 pandemic, with a shift from 9 to 42 countries affected between March and May 2020 [5]. Factors associated with the rapid spread of COVID-19 virus in the region included poor public health infrastructure, underreporting and limited testing resources [6]. To mitigate the impact of COVID-19 in low resource settings, continuous assessment of implemented public health measures as well as healthcare resource capacity must be pursued to prevent overwhelming the healthcare system [7]. Some of the public health measures implemented by the Nigerian federal government included initiated of a presidential task force to provide a high-level strategic national response; launched public health awareness campaigns; testing, contract tracing and isolation of all COVID-19 confirmed cases; air travel ban and local lockdown of three most affected states [4].

Predicting COVID-19 population spread and healthcare resource needs for symptomatic patients using models is essential to ensure the health system will continue to operate efficiently during the pandemic, minimize morbidity and reduce society disruption [8]. Simulation models can help to inform healthcare resource needs under different scenarios to improve planning and support procurement strategies, especially in resource-limited settings [9]. Nigeria, like many African countries, has limited healthcare resources and infrastructure. Before the start of the pandemic, Nigeria had an estimated 0.2 hospital beds per 1,000 population, 350 intensive care unit (ICU) beds without ventilators (equivalent to 0.07 ICU beds per 100,000 population) and 450 ventilated beds [10-12]. With the country´s cumulative COVID-19 cases at 54,588 on September 3^rd^, 2020 [4] and case numbers still increasing, forecasting how COVID-19 will affect hospital resources under different epidemic trajectories and resource scenarios are critical for Nigeria´s COVID-19 response. Our objective is to predict the short-term effect of COVID-19 on hospital resources in Nigeria for a range of COVID-19 epidemic and hospital capacity scenarios using a health system simulation model.

## Methods

**Study design:** we adopted the previously published COVID-19 Resource Estimator (CORE) model from Ontario, Canada [13], to fit the healthcare system and COVID-19 epidemiology in Nigeria. Our primary outcomes included projected cumulative number of COVID-19 cases, number of days until depletion of ward bed and ventilated ICU bed resources and number of avoidable deaths assuming no resource constraint. Outcomes were predicted over a 60-day time horizon in daily time steps.

**Figure 1:**
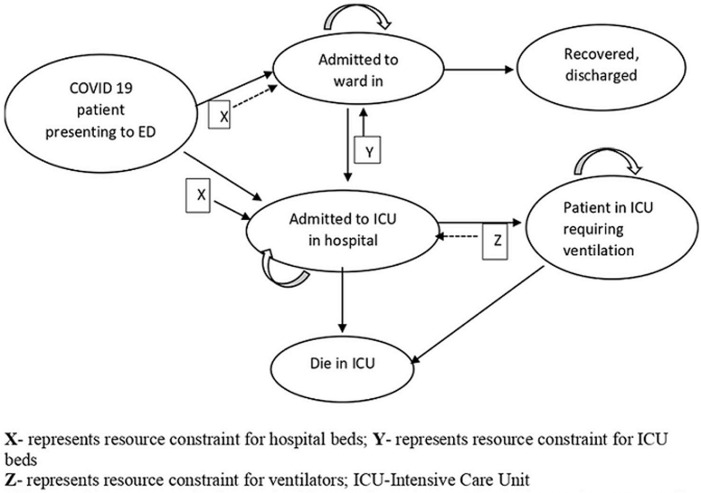
CORE model schematic

**CORE model structure:** we used the CORE model that was available as an interactive software application online [11]. A detailed description of the CORE model is provided elsewhere [9]. Briefly, CORE is a discrete-time, individual-level, health-state transition model of symptomatic COVID-19 patients. CORE simulates a dynamic population of symptomatic COVID-19 adult patients (18 years and above) who present to the emergency department (ED), where they are either sent home to self- isolate or admitted to the hospital. If admitted, COVID-19 patients are assigned to either a general ward or ICU, depending on disease severity. Seventy-eight percent of ICU patients were assumed to require invasive mechanical ventilation [9,10]. CORE model is presented as Figure 1.

**CORE model assumptions:** several assumptions were made in the CORE model. For simulated patients in the model, resource needs were based on the patient´s health state (i.e. in a general ward or ICU). If there was a lack of ward or ICU beds, patients remained with their current available resources until the needed resource became available. Patients requiring mechanical ventilation but did not have access were assumed dead within 24 hours. Mortality risk was attributed to only ICU patients with or without a need for a ventilator. Patients awaiting an ICU bed (but not mechanical ventilation) were assumed to have the same risk of death as patients in ICU. Ward beds were assumed available upon patients´ recovery. ICU beds with/without ventilators were assumed to be freed up upon recovery or death of patients. Available resources were prioritized based on the patient´s location (e.g. ward beds will be prioritized to ICU patients before new admissions) and the length of waiting time of a patient since admission. The CORE model assumed that other essential resources, including personal protective equipment and essential medications, would be available in sufficient quantities. It also assumed all available beds are adequately staffed and no staffing shortage would emerge with vast-increase in hospital resources.

**Assumptions specific to Nigeria hospital setting:** we assumed that COVID-19 patients would only be treated in hospitals licensed by the Nigeria Centre for Disease Control [14]. Patients were assumed to be admitted to ICU only when in need of mechanical ventilation and that all ICU beds are vented.

**Model parameters:** all model parameters are listed in Table 1. COVID-19 acute resource utilization; the fixed parameters in the CORE model included probabilities of hospital and ICU admission obtained from reported Canadian COVID-19 data, reflecting clinical need without resource constraints [9,15]. Mean length of hospitalization and the probability of death for COVID-19 patients in the ward and ICU were based on reported data for patients admitted for moderate acute respiratory distress syndrome (ARDS), based on a similar clinical manifestation [9,16-18]. The proportion of ICU patients requiring mechanical ventilation was obtained from reported Canadian data [9,16].

**Table 1:** CORE model parameters

Parameter	Value
Number of ward beds (pre- COVID-19)	0.2 per 1,000 population
Number of ICU beds (pre- COVID-19)	350
Number of ventilators (pre- COVID-19)	450
Number of new COVID-19 cases on September 3<sup>rd</sup>, 2020	125
Cumulative number of COVID-19 cases reported until September 3<sup>rd</sup>,2020	54,588
Probability of needing admission to hospital	0.18
Probability of needing ICU-level care given admission to hospital	0.48
Probability of patients in ICU needing ventilation	0.78
Probability of patients on the ward who deteriorate and need ICU-level care	0
Length of stay, ward (no ICU admission)	17 days
Length of stay, ICU (with or without ventilation)	11 days
Length of stay, ward after a stay in ICU	6 days
Probability of death, ward patients	0
Probability of death, ICU patients (with/without ventilation)	0.35
Probability of death, patients waiting for ventilation	1.0

ICU: intensive care unit; NCDC: Nigeria centre of disease control

**Figure 2:**
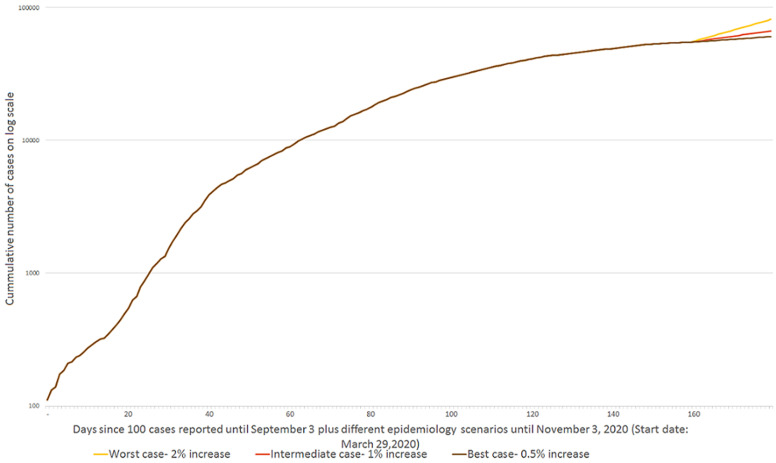
cumulative cases of COVID-19 using different epidemic trajectories

Epidemic trajectories: we used reported data on daily and cumulative COVID-19 cases up to September 3^rd^, 2020, when the number of cumulative cases was 54,588, as published by the Nigeria Centre for Disease Control (NCDC) [4]. We forecasted three scenarios: best case, intermediate case and worst case - to predict the trajectory of COVID-19 cases in Nigeria for 60 days, starting with data from September 3^rd^, 2020. In the three scenarios, we started our prediction with 54,588 confirmed COVID-19 cumulative cases and 125 new cases within 24 hours. For our best-case scenario, we assumed an infection growth rate of 0.5% (i.e. 50% reduction in daily infection growth rate from September 4^th^, 2020) to depict continuous implementation of aggressive public health measures (i.e. social distancing, school closure and travel restrictions). To our knowledge, no African country has a daily infection growth rate of 0.5% from the day when 100 cases were reported. In our intermediate scenario analysis, we used a 1% infection growth rate, which is the average growth rate from the last two months (August 3^rd^, 2020 to September 3^rd^, 2020) in Nigeria [4]. For our worst-case scenario, we assumed an infection growth rate of 2%, which suggests a scenario where implemented public health measures are rapidly weakened, economy re-opening is fast-tracked and broad community transmission is ongoing. Infection growth rates across countries were calculated using daily reported COVID-19 cases from the John Hopkins University repository [19]. We did not assume an epidemic peak in our study since our time horizon is short (60 days) and the current epidemic trajectory in Nigeria suggests the epidemic growth has peaked and the number of daily new cases is declining. Case predictions for the three scenarios are shown in Figure 2 (the cumulative daily number of cases are provided in supplementary material).

Resource capacity scenarios: we obtained data on the total number of ward beds, intensive care unit beds and ventilators before the pandemic from the published literature and press reports [10-12]. Our data estimate for total ICU beds and ventilators were obtained from a current press report by Nigeria health officials assessing the available health resources in Nigeria. We modelled three resource scenarios: conservative, expanded and surplus. For the conservative scenario, we assumed that 50% of ventilated ICU beds (175/350) and 25% of hospital beds (10,000/40,000) are available to treat symptomatic patients based on recent press reports and a national survey conducted by a pharmaceutical company [10-12]. In the expanded resource scenario, we added 200 ventilated ICU beds to the proportion available for treating COVID-19 ill-patients in conservative resource scenario and an additional 10,000 ward beds to the proportion of ward beds available in the conservative resource scenario. The assumption was based on reduced clinical activity enabling re-allocation of privately-owned hospital resources, the recent increase in health resource supplies from donors and the increase in government investment in healthcare services and resources since the start of the pandemic [20]. For the surplus scenario, we assumed an additional 1,000 ventilated ICU beds to the proportion available for treating COVID-19 ill-patients in expanded resource scenario and an extra 15,000 ward beds to the proportion of ward beds in expanded scenario resources based on potential funding from foreign donors, high-income countries and international health organizations [21]. In the three hospital resource scenarios, we assumed adequate hospital staffing based on the recall of retired health personnel to service by the Nigeria federal government and the ongoing recruitment and training of volunteers across the country to boost staffing capacity [22,23].

## Results

For the best case epidemic trajectory (0.5% growth rate), our study predicted 73,631 COVID-19 cases over 60 days (Figure 2, Annex 1) of whom 1,308 patients would require hospital admission to ward and ventilated ICU beds. In all resource scenarios, ward bed and ventilated ICU bed would not be depleted before 60 days (Table 2). For the intermediate epidemic trajectory (1% growth rate), we predicted 99,170 cumulative cases (Annex 1) and 1,492 hospital admissions to ward and ventilated ICU beds over the 60-day time horizon. There will be no shortage of ventilated ICU beds and ward beds in all three resource scenarios.

**Table 2:** estimated time to resource depletion by resource and epidemic trajectory scenarios

		Estimated time to resource depletion (in days)		Number of avoidable deaths
		Ward beds	Ventilated ICU bed	
**Resource scenario: conservative**				
Epidemic scenario	Best case (0.5% increase)	NR	NR	NR
	Intermediate (1% increase)	NR	NR	NR
	Worst-case (2% increase)	NR	39	16
**Resource scenario: expanded**				
Epidemic scenario	Best case (0.5% increase)	NR	NR	NR
	Intermediate (1% increase)	NR	NR	NR
	Worst-case (2% increase)	NR	NR	NR
**Resource scenario: surplus**				
Epidemic scenarios	Best case (0.5% increase)	NR	NR	NR
	Intermediate (1% increase)	NR	NR	NR
	Worst-case (2% increase)	NR	NR	NR

ICU: intensive care unit; NR: no resource constraint will be experienced within the next 60 days

For the worst-case epidemic trajectory (2% growth rate), we predicted 179,105 COVID-19 cases and 2,178 hospital admissions to ward and ventilated ICU beds over 60 days. In all three resource scenarios, ward beds would not be depleted. In the conservative resource scenario, ventilated ICU bed would be unavailable after 39 days. However, by increasing the ventilated ICU bed supply as assumed in the expanded and surplus resource scenarios, ventilated ICU beds would be available over the time horizon. Across all scenarios, sufficient resources to care for hospitalized COVID-19 patients would be unavailable in worst case epidemic trajectory (i.e. 2% infection growth rate) assuming the conservative resource scenario. Assuming the conservative resource scenario, the worst-case epidemic trajectory projects 16 preventable deaths over 60 days. In the expanded and surplus resource scenarios, assuming all three epidemic trajectories, no death due to the unavailability of resources was predicted to occur over 60 days.

## Discussion

We demonstrated that an increase in healthcare resources is essential for Nigeria´s health system to care for COVID-19 hospitalized patients, even at low infection growth rates. Maintaining aggressive public health measures is crucial in order to continue to lower community spread and prevent hospital resource constraints. By September 8^th^, some public health measures implemented in Nigeria, such as domestic and international air travel ban, had been eased with the country´s average infection growth rate at 4% (averaging from March 29^th^ when 100 cases were reported to September 3^rd^, 2020). During the early days of the pandemic in Nigeria, the federal government implemented an early ban on international travel as well as effective contact tracing and testing strategies in the most affected states. These public health measures have proven to be successful and contributed to the decline in the average infection growth rate to 1% within the last two months. While the federal government acted promptly to initiate a local inter-state lockdown on March 31^st^, 2020, this decision was reversed in May due to economic pressures, despite the fluctuating low to the high daily number of cases [24], however, other public health measures such as air travel ban, universal masking were maintained for a more extended period. Our analysis shows that maintaining strong public health measures for a long time with a substantial increase in resources is crucial to curtailing COVID-19 spread and prevent hospital resource constraints and health system collapse in Nigeria. Our findings are supported by modelling studies that predict an expanding increase in the number of COVID-19 cases in the Africa region [25] and the need for strong public health measures, abundancy of medical supplies and population behavioural change to mitigate the spread of COVID-19 in the region [26].

Our study has some limitations. Our estimated number of ward beds was based on a national survey by a pharmaceutical company published in 2007 and might not represent the country´s current capacity. While keeping with the current literature, we assumed that death would only occur in critically ill patients, which may underestimate mortality. Since the start of the pandemic, recruitment and training of new hospital staff have been ongoing, but actual data on the number of recruited staff was not available at the time of modelling, limiting our knowledge on the effect of vast expansion of hospital resources on staff capacity. Our study relies on reported cases to forecast future epidemic trajectories. The model does not account for underreporting of daily cases and long-time lag in case reports, which are common problems that occur in low resource settings due to limited testing capacity [6]. Due to the unavailability of detailed COVID-19 data, some parameters included in the model were estimates obtained from a study on ARDS, a disease with similar clinical manifestations with COVID-19. Our healthcare resource utilization probabilities parameters (i.e. length of hospital stay and probability for need of ward, ICU and ventilator admissions and probability of death) were estimates from Canadian setting. While these parameter values arguably deviate from observed data in Africa, they are likely estimates for COVID-19 disease severity.

Our study has several strengths. We incorporated observed incidence data from Nigeria and other Africa countries with a range of epidemic trajectories to strengthen the validity of our epidemic predictions. The CORE model considers resource constraints within the health system and estimated deaths due to resource depletion. We stratified mortality between patients who receive adequate care in ICU and those who did not to estimate the number of patients who will likely die from an overwhelmed health system.

The COVID-19 epidemic trajectory is slower in Africa [6], with many countries within 5-10% average infection growth rates since 100 cases reported to September 3^rd^, 2020. Our epidemic trajectory and resource scenario predictions could apply to other low resource setting countries, especially in the Africa region. Our resource scenarios estimates could also be used as a guide to inform pandemic preparedness planning and policy development.

## Conclusion

The epidemic trajectory of COVID-19 appears to have a low growth rate and the implemented public health measures have helped to reduce community spread in Nigeria; however, more hospital resources in Nigeria are required to manage the daily number of COVID-19 critically ill-patients. To mitigate the impact of COVID-19, implementing more aggressive public health measures is vital and strategies to exponentially increase the number of health resources available in the country need to be put in place to prevent overwhelming the healthcare system.

### What is known about this topic

- While the COVID-19 trajectory remains low in Africa, there is still an increasing community spread of the virus in many African countries;
- Limited healthcare resources available in the region may not be sufficient to cope with increasing numbers of COVID-19 cases.

### What this study adds

- Using the COVID-19 Resource Estimator (CORE) model, we demonstrate that implementing and maintaining aggressive public health measures to keep the epidemic growth at a very low rate is essential;
- A substantial increase in healthcare resources is critical to minimize the impact of COVID-19 on morbidity and mortality in Nigeria.

## Annex

**Annex 1**: epidemic predictions between September 3^rd^, 2020 and November 3^rd^, 2020 (PDF-209Kb)
